# Intestinal obstruction in pregnancy—a rare presentation of uterine perforation

**DOI:** 10.1186/s12884-023-05827-8

**Published:** 2023-07-11

**Authors:** Jiayu Shen, Xinyuan Teng, Jing Chen, Ligui Jin, Liquan Wang

**Affiliations:** Department of Obstetrics, The Second Affiliated Hospital of Zhejiang University School of Medicine, Zhejiang Province, Hangzhou, China

**Keywords:** Acute intestinal obstruction, Uterine rupture, Pregnancy

## Abstract

**Background:**

Intestinal obstruction is an uncommon non-obstetric condition during pregnancy which may cause maternal and fetal mortality. Clinicians are confronted with challenges in diagnosis and treatment of intestinal obstruction due to the overlapping symptoms, concerns over radiological evaluation, and surgical risks.

**Case presentation:**

We reported a 39-year old, gravida 7, para 2, woman who suffered from acute intestinal obstruction at 34 weeks of gestation. Ultrasonography and abdominal computed tomography were applied for intestinal obstruction diagnose. Conservative treatment was initially attempted. But following ultrasound found the absence of fluid in the amniotic sac and the patient showed no improvement in clinical symptoms. An emergency caesarean section was then performed. Intra-operative assessment showed dense adhesion between the left wall of uterus and omentum, descending colon, and sigmoid colon. After adhesion dialysis, uterine rupture with complete opening of the uterine wall at the site of left uterine cornua was found without active bleeding. The uterine rupture was then repaired.

**Conclusions:**

Although uncommon during pregnancy, clinical suspicion of bowel obstruction is necessary especially in women with a history of abdominal surgery. Surgical intervention is indicated when conservative therapy fails and when there are signs of abnormal fetal conditions and worsened symptoms.

## Background

The diagnosis and management of acute abdominal pain in pregnancy is a challenging task as multiple conditions can be accompanied with similar symptoms, including obstetric causes and non-pregnancy-related causes. Obstetric conditions include ectopic pregnancy, septic abortion, placental abruption, chorioamnionitis, premature labor, and uterine rupture [1]. The common non-obstetric causes seen in pregnancy are appendicitis, cholecystitis, gastroenteritis, and pancreatitis [2–7]. Intestinal obstruction in pregnancy (IOP) is rare with reported incidence rates of 0.001–0.003% [8]. The commonest cause of IOP is post-surgical adhesions. The growing uterus can lead to the physiological and anatomical changes of gastrointestinal tract, which may worsen obstruction [9]. Despite being rare, IOP is associated with significant maternal and fetal mortality. Thus, the appropriate and timely diagnosis and treatment of intestinal obstruction complicating pregnancy is of vital importance. Here we present a unique and interesting case of intestinal obstruction complicating with uterine rupture in a female in her third trimester of pregnancy.

## Case presentation

A 39-year-old, gravida 7, para 2, woman came to the local hospital with a complaint of lower abdominal dull pain for 3 h at 34 weeks’ gestation. Her obstetrical history began with two preterm spontaneous vaginal delivery (one at 28 weeks’ gestation; one at 30 weeks’ gestation) at the age of 18 and 19, respectively. Her third, fourth, and fifth pregnancies ended in abortion at early gestation with dilation & curettage treatment. In her previous pregnancy at 36 years of age, she received laparoscopic left salpingectomy and methotrexate (MTX) treatment due to ectopic pregnancy. No other surgeries on the pelvis or abdomen has been performed.

Her general condition was stable. Physical examination showed normal secretions, no vaginal bleeding or fluid, no cervical dilation, but irregular preterm contractions with a shortened cervical length. Ultrasonography revealed a decreased amniotic fluid index (AFI 4.0 cm) and a single intrauterine pregnancy with breech presentation and positive fetal heart activity. The placenta was located on the anterior wall of the uterus. Laboratory tests were performed at the time of admission and did not show any significant findings, and hemoglobin level was 120 g/L. Due to the risk of premature birth, dydrogesterone was used to relieve uterine contraction and dexamethasone was administered to accelerate fetal lung maturation. Over 2 days following admission, her symptoms gradually worsened. She presented with diffuse abdominal pain and distension. She also reported nausea and had problem in defecating. Ultrasonography of the abdomen represented intestinal dilatation and multiple intestinal contents and air-fluid levels in the colon. Therefore, an intestinal obstruction was suspected. Enema was used to help intestinal peristalsis.

**Fig. 1:**
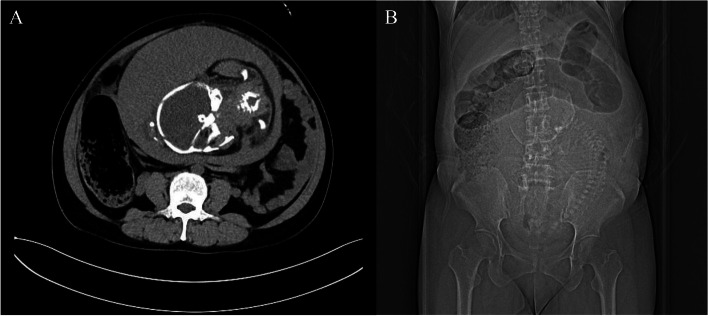
Axial (**A**) and coronal (**B**) views of computed tomography (CT) of the abdomen and pelvis. CT demonstrated gross distension of ascending colon and transverse colon

**Fig. 2:**
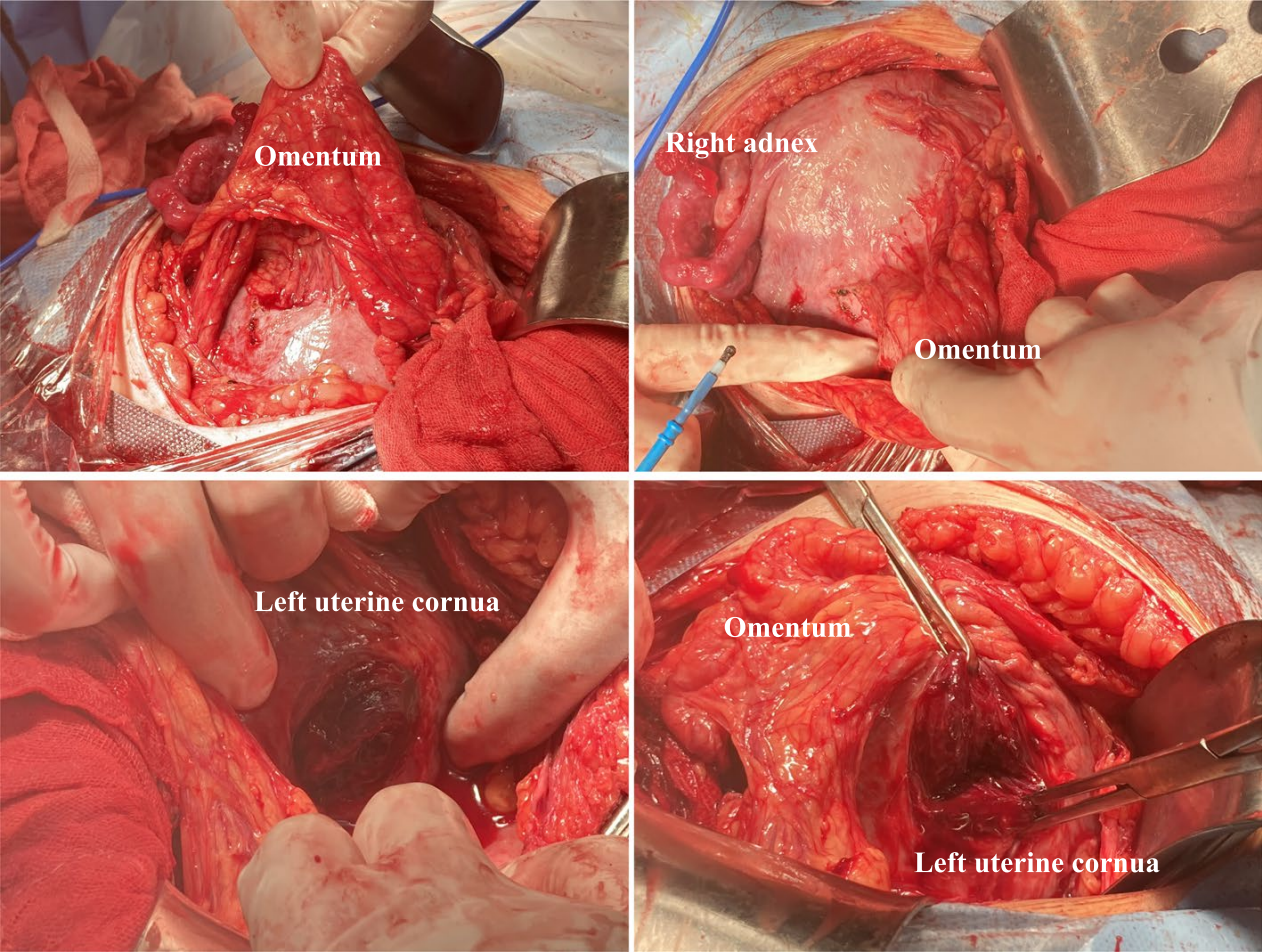
Images of uterus and omentum taken during the emergency C-section. Dense adhesion was found between the left wall of uterus and omentum. After adhesion dialysis, signs of uterine rupture at the site of left uterine cornua was found without active bleeding

As there was no improvement in her condition, the patient requested a transfer to our hospital. Clinical examination revealed epigastric tenderness and mild abdominal distension, with no signs of guarding or peritonitis. Bowel sounds were weak. Consulting with the surgeon on duty, an abdominal computed tomography (CT) was performed after informed consent from the patient. CT represented dilatation of ascending colon and transverse colon (Fig. 1). The symptoms were relieved after a nasogastric tube was inserted initially and enema was used. Since ultrasonography revealed oligohydramnios, a test for amniotic fluid crystal was performed, showing no signs of premature rupture of fetal membrane. On the fourth day following admission in our hospital, ultrasonography showed merely no fluid in the amniotic sac and the patient complained of no complete relief of bowel obstruction. Therefore, an emergency caesarean section (C-section) was performed. A healthy baby was delivered with no complications. Intra-operative assessment showed dense adhesion between the left wall of uterus and omentum, descending colon, and sigmoid colon. After adhesion dialysis and intestinal arrangement performed by a colon and rectal surgeon, uterine rupture with complete opening of the uterine wall at the site of left uterine cornua was found without active bleeding (Fig. 2). The uterine scar was repaired using a double-layer closure.

**Fig. 3:**
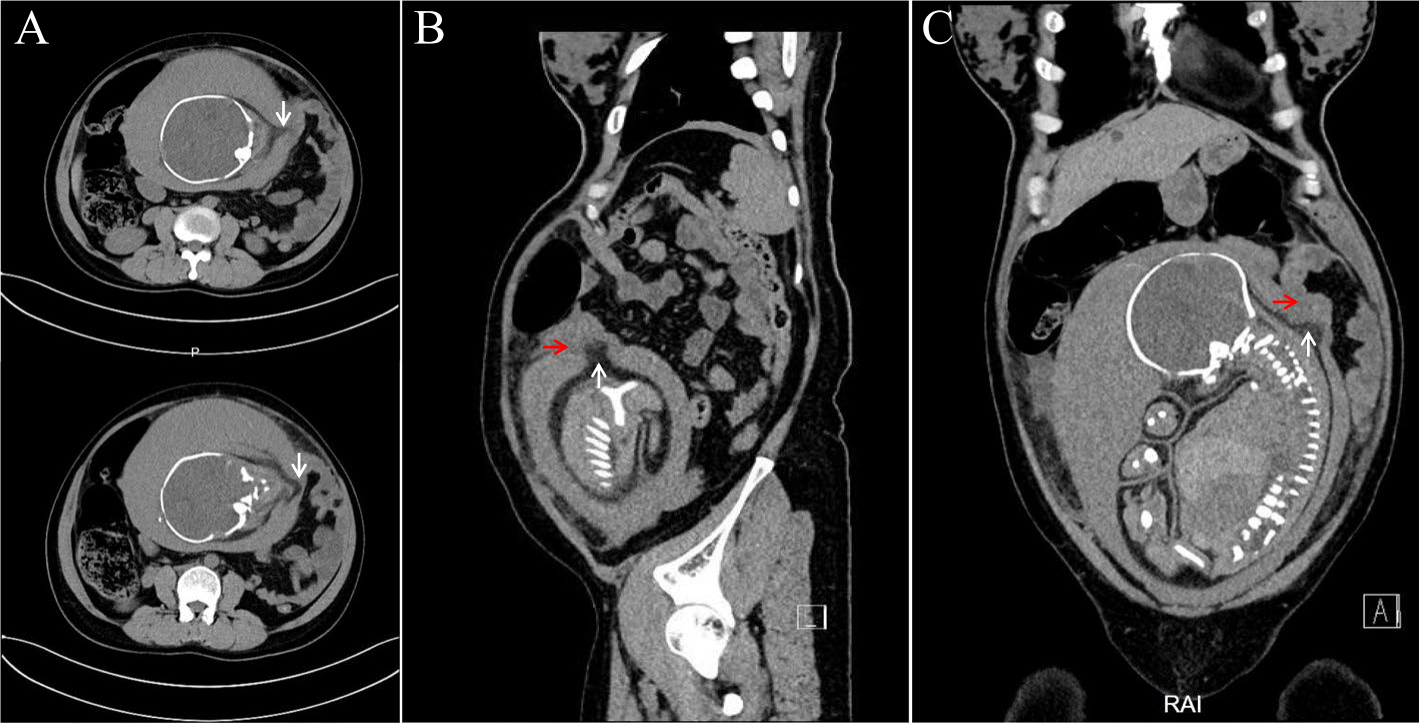
Axial (**A**), sagittal (**B**), and coronal (**C**) views of CT of the uterus. CT showing discontinuity of the uterine muscle layer on the left cornua (white arrow) and adhesion between the ruptured site and omentum (red arrow)

Re-evaluation of the former abdominal CT was taken, revealing discontinuity of the uterine muscle layer in the left uterine cornua (Fig. 3), which was consistent with intraoperative findings. Considering that the hemoglobin level didn’t decrease, her blood pressure remained stable, and no fetal distress happened during her labor of the birth, we assumed that the uterine rupture may occurred very early. Since omentum and colon were attached to the site of perforation, no clinical signs of uterine rupture were presented. As uterus enlarged, changes in the transit of intestinal tract decreased the intestinal peristalsis and then caused obstruction.

The patient recovered well without any complications and was discharged on the fifth postoperative day.

## Discussion

It is of paramount importance to appropriately diagnose and treat intestinal obstruction complicating pregnancy as it is associated with significant maternal and fetal mortality. IOP remains the second most common non-obstetric condition for surgical intervention during pregnancy, usually occurring in the third trimester. Reported causes of IOP include postsurgical adhesion, volvulus, intussusceptions, hernia, carcinoma, and idiopathic ileus, of which postsurgical adhesion is the commonest cause. Adhesive obstruction occurs more frequently in advanced pregnancy with 6%, 28%, 45% during the first, second, and third trimester, respectively [10]. In this case, it seems to be secondary to old uterine perforation which may be secondary to multiple dilation & curettage and left salpingectomy, leading to adherence of gut at the time of perforation. In the advanced stage of pregnancy, the growing uterus made changes in the transit of intestinal tract which finally decreased the intestinal peristalsis and caused obstruction.

Diagnosis of IOP can be difficult to make. Clinical symptoms are often attributed mistakenly to pregnancy and physical examination is often challenging as the gravid uterus poses limitations to proper examination of the abdominal quadrants. Besides, the maternal may be reluctant to take plain films owing to the risk of ionising radiation. Previous literature reviews have demonstrated that radiological methods are acceptable in pregnancy since a single plain abdomen X-ray is around 100 millirads and an abdomen CT scan is around 3.5 rads [11–14]. Fetal risk of anomalies, abortion, or growth restriction have not been reported with radiation exposure of less than 50 mGy [11]. Besides, abdominal radiography can accurately diagnose intestinal obstruction and quickly determine whether intestinal perforation has occurred. CT owns greater sensitivity and specificity than plain radiography and can reliably determine the cause of obstruction and associated complications, which assists in surgical planning [12]. And CT has been recommended by the American College of Radiology as the initial imaging modality for evaluating intestinal obstruction in patients with clinical suspicion [13]. Thus, diagnostic imaging utilizing ionising radiation should be considered during pregnancy when the benefits outweigh the risks. Magnetic resonance imaging (MRI), which is capable of multiplanar imaging and excellent soft tissue contrast, can avoid the risk of ionising radiation and present a useful tool for diagnosing IOP [14]. However, the high cost and technical expertise and time required to perform MRI restricts its clinical application. In the emergently presenting patient, abdominal ultrasound is also a preferred modality in evaluating dilated bowel loops and abnormal peristalsis.

The principle of management of intestinal obstruction in pregnant women is similar to non-pregnant states [14, 15]. Conservative approaches should be applied initially, such as nasogastric decompression, intravenous fluid, and intravenous antispasmodics. Surgical management is indicated when conservative intervention fails and when there are signs of fetal distress and signs of bowel ischemia or necrosis. In this case, nonoperative management was initially attempted. However, the absence of amniotic fluid and the persistent obstruction symptoms made the final decisive elements in the decision to perform an emergency C-section. The etiologies of oligohydraminos include premature rupture of membranes, placental insufficiency, or congenital urogenital anomalies [16–18]. In this case, the patient denied that she presented with leaking of fluid from the vagina, or the sensation of wetness at the vagina or perineum. And no urogenital anomalies were indicated during routine examination. We assumed that the absence of the amniotic fluid may be caused by the rupture of the uterus. The amniotic membrane at the perforation site is structurally altered and easily disrupted, leading to a loss of amniotic fluid in the abdomen which may be consequently absorbed by the peritoneum. Both the absence of amniotic fluid and the presence of adhesion can make the C-section procedure and the fetal extraction became more difficult. Dense adhesions alter the usual structures and increase the risk of complications, such bowel or bladder injury, and excessive blood loss [19]. Thus, when a difficult C-section is expected, a multidisciplinary team should be available.

## Conclusion

Intestinal obstruction is an extremely rare event during pregnancy but is associated with significant maternal and fetal mortality. It is commonly caused by adhesive bands from previous abdominal surgeries. Obstetricians should be highly vigilant about the diagnosis of bowel obstruction especially in women with a history of abdominal surgery. Diagnostic modalities, including ultrasound, X ray, CT, and MRI, may be offered to pregnant women. Conservative therapy can be initially tried, but surgical intervention must be timely performed when conservative management fails and when there are signs of abnormal fetal conditions and worsened symptoms.

## Data Availability

Not applicable.

## Abbreviations

IOP: Intestinal obstruction in pregnancy
MTX: Methotrexate
AFI: Amniotic fluid index
CT: Computed tomography
C-section: Caesarean section
MRI: Magnetic resonance imaging
